# Glycoproteomic analysis identifies human glycoproteins secreted from HIV latently infected T cells and reveals their presence in HIV^+^ plasma

**DOI:** 10.1186/1559-0275-11-9

**Published:** 2014-03-06

**Authors:** Weiming Yang, Jian-Ying Zhou, Li Chen, Minghui Ao, Shisheng Sun, Paul Aiyetan, Antoine Simmons, Hui Zhang, Jay Brooks Jackson

**Affiliations:** 1 Department of Pathology, Johns Hopkins University School of Medicine, 1550 Orleans Street, CRBII, Room 3 M-03, Baltimore MD 21205, USA

**Keywords:** Glycoproteomics, SPEG, HIV, T cells, Medium, Plasma

## Abstract

Glycoproteins secreted into plasma from T cells infected with human immunodeficiency virus (HIV) latent infection may provide insight into understanding the host response to HIV infection *in vivo*. Glycoproteomics, which evaluates the level of the glycoproteome, remains a novel approach to study this host response to HIV. In order to identify human glycoproteins secreted from T cells with latent HIV infection, the medium from cultured HIV replication-competent T cells was compared with the medium from cultured parental A3.01 cells via solid phase extraction of glycopeptides (SPEG) and high-performance liquid chromatography-tandem mass spectrometry (HPLC-MS/MS). Using these methods, 59 human glycoproteins were identified as having significantly different abundance levels between the media from these two cell lines. The relevance of these 59 proteins to HIV infection *in vivo* was assessed in plasma from HIV^+^ and HIV^-^ subjects. Comparison between T cell and plasma revealed that six glycoproteins (galectin-3-binding protein, L-selectin, neogenin, adenosine deaminase CECR1, ICOS ligand and phospholipid transfer protein) were significantly elevated in the HIV^+^ T cells and plasma studies. These findings suggest that the response of T cells harboring latent HIV infection contributed, in part, to the glycoprotein changes in HIV^+^ plasma. These proteins, once validated, could provide insight into host-HIV interaction.

## Introduction

In 2011, there were approximately 34 million individuals living with HIV [1]. In the same year, it is estimated that there were approximately 2.5 million new infections and 1.7 million AIDS related deaths [1]. Although tremendous efforts have been devoted to prevent HIV transmission, to date, no effective HIV vaccine is available [2]. Instead, highly active antiretroviral therapy (HAART) is routinely used to treat patients infected with HIV and it has been shown to be effective in the prevention of HIV transmission [3]. HAART has greatly improved the lifespan and quality of life of the HIV infected population [4]. However, HIV related illnesses such as neurodegenerative diseases, cancers, and bacterial infections have emerged as important issues in HIV^+^ patients [5-8]. To identify proteins in a complex sample for the (early) detection, diagnosis, prognosis, treatment prediction and the elucidation of the pathogenesis of these diseases, proteomic studies have demonstrated their applicability in a high throughput fashion [9-11]. However, the urgent need for the identification of biomarkers of HIV and its related diseases remains unmet [8].

HIV is a *lentivirus* which belongs to the retrovirus family [12]. It primarily infects immune cells containing CD4 receptors, such as helper T cells, dendritic cells (DC) and macrophages, and establishes latent infection in these cells [13]. An inevitable step in the viral replication cycle is the integration of viral DNA into the T cell genome prior to either latent infection or active production of virions [14]. The infection of the immune cells, which reside in the blood and draining lymph tissues, results in the atypical secretion and shedding of disease-related proteins into the peripheral blood [15]. Thus, the blood of patients infected with HIV is considered to be an excellent source to identify proteins that are associated with HIV infection. Studies using proteomic approaches that aim to discover new biomarkers of HIV and its related diseases have identified protein abundance changes in plasma and serum [9,10,16-19].

Glycoproteomics, a sub-proteomic approach that specifically investigates the levels of glycoproteins in a complex sample, has been widely applied for cancer biomarker discovery [20-22]. This approach is promising because glycoproteins are ubiquitous in extracellular secretion, are known to be dysregulated in cancerous cells, and are likely to enter the bloodstream, making them attractive candidates for cancer biomarkers [23]. The significance of glycoproteins as biomarkers has shown great promise in that most of the U.S. Food and Drug Administration (FDA) approved cancer biomarkers are glycoproteins. These biomarkers include prostate-specific antigen (PSA) for prostate cancer, CA125 for ovarian cancer, and CA15-3 for breast cancer [24,25]. In HIV infection, the host systemic reaction to infection of immune cells, inflammation, chronic activation, repeated evasion-recognition and reconstitution of immune system likely triggers the secretion or shedding of a large number of glycoproteins into the bloodstream [26,27]. It has been observed that the global and HIV-specific antibody-glycosylation profile is altered during HIV infection [28]. It is possible that the aberrant presence of glycoproteins other than antibodies may be specifically associated with HIV infection given the unique immune cell tropism of the virus [13]. However, the change in glycoproteins caused by HIV infection has not been determined in previous studies and remains unclear.

In the blood, N-linked glycosylated proteins are particularly interesting because N-linked glycosylation is a designated signal for the extracellular secretion of proteins [15,22]. To study the N-linked glycoproteins in a high throughput manner, we previously developed a method for the hydrazide chemistry-based solid phase capture of glycopeptides and coupled it with the use of PNGase F for the specific release of ‘formerly’ N-glycosylated peptides (only the peptides originally attached to glycans) [29-31]. The isolated formerly N-glycosylated peptides are analyzed by LC separation followed by tandem mass spectrometry (MS/MS), which allows the high throughput identification and quantification of a large number of glycoproteins [32-34]. This feature of the technique provides a unique opportunity to detect protein changes in plasma-derived immune cells [35,36].

In this study, LC-MS/MS based quantitative glycoproteomics was used to examine the altered levels of glycoproteins associated with HIV latent infection in T cells (ACH-2 cell lines) and plasma from HIV^+^ patients [29,30]. The advantage of label-free LC-MS-MS/MS based quantitative glycoproteomics is the ability to compare an unlimited number of samples with a deeper coverage of identification compared to strategies that incorporate stable isotope labeling [37,38]. The current study started with the identification of dysregulated glycoproteins from the media of T cell lines with HIV latent infection to determine putative glycoprotein candidates, followed by the use of HIV^+^ plasma to identify the glycoprotein changes that could be influenced by the aberrant secretion of glycoproteins from the T cells harboring HIV DNA in their genomes. To our knowledge, this is the first study to identify glycoproteins that are potentially associated with HIV infection in plasma by high throughput glycoproteomics using HIV^+^ T cells and plasma.

## Methods

### Chemicals

The bicinchoninic acid (BCA) protein assay kit was purchased from Pierce (Rockford, IL); hydrazide support and sodium periodate were purchased from Bio-Rad (Hercules, CA); sequencing-grade trypsin was purchased from Promega (Madison, WI); PNGase F was purchased from New England Biolabs (Ipswich, MA); Sep-Pak C18 1 cc Vac Cartridges were purchased from Waters (Milford, MA); and acetonitrile (ACN), trifluoroacetic acid (TFA), formic acid, urea, tris(2-carboxyethyl)phosphine (TCEP), iodoacetamide, phorbol 12-myristate 13-acetate (PMA), 5 M NaCl, sodium dodecyl sulfate (SDS), 10× phosphate buffered saline buffer (PBS) pH 7.4 and 1 M Tris–HCl pH 8.0 buffer were purchased from Sigma-Aldrich (St. Louis, MO).

### Patient samples

Plasma was tested for HIV antibody and HIV RNA viral loads were determined if the plasma tested positive for HIV. The plasma was aliquoted and immediately stored at -80°C until use (Table 1). Prior to the study, samples were de-identified. The Johns Hopkins Medicine Institutional Review Board (IRB) approved the use of the samples.

**Table 1:** Demographics and viral loads of patients

**Plasmas**	**Age, years (mean, SD)**		**Sex, male (N, %)**		**Viral load**
HIV-(n=10)	48.5	±14.8	5	50%	ND
HIV+(n=10)	40.4	±13.6	6	60%	<400 to 188,412
					<400
					<400
					188,412
					38,254
					689
					<400
					106,239
					<400
					<400
					3,915

(ND-not determined).

### Cell culture

The HIV-1 latently infected T cell line ACH-2 and its parental cell line A3.01 were obtained from Dr. Thomas Folks through the AIDS Research and Reference Reagent Program, Division of AIDS, NIAID, NIH [39-41]. The ACH-2 cell line is a sub-clone of the human A3.01 T cell line derived from acute infection with the LAV strain of HIV-1. The HIV DNA is integrated into the genome of the T cell line. Low levels of supernatant RT and p24 antigen are constitutively produced in ACH-2 cells. HIV-1 replication can be induced with phorbol 12-myristate 13-acetate (PMA) or IL-2 to produce high levels of infectious HIV-1. Thus, the cell line is HIV replication competent. However, we did not stimulate the cells with PMA or IL2 in the study. ACH-2 and A3.01 cells were cultured in RPMI 1640 supplemented with 2 mm L-glutamine (Gibco Laboratories, Grand Island, NY), 100 U/ml penicillin, 100 g/ml streptomycin (Invitrogen, Carlsbad, CA) and 10% (v/v) heat-inactivated fetal calf serum (FCS, Hyclone Laboratories, Logan, UT). In this study, cells were pelleted and washed in serum free medium 5 times and resuspended in serum free medium. After 48 hours of incubation at 37°C in 5% CO_2_, the cells were pelleted by low speed centrifugation and the medium was harvested, filtered through a 0.22 μm filter unit and concentrated using a 3 kDa cut-off centrifugal filter unit (Millipore, Bedford, MA). The concentrated medium was diluted in 800 μl of 8 M urea in 0.2 M Tris–HCl with 0.1% (w/v) SDS solution (pH 8.0). Protein concentration was determined by BCA assay.

### N-linked glycopeptide isolation

Cell culture media (1 mg protein) and plasma (20 μl containing approximately 1.6 mg protein) were added to 8 M urea in 0.2 M Tris–HCl with 0.1% (w/v) SDS solution (pH 8.0). Proteins were reduced with 10 mM TCEP in Tris–HCl pH 8.0 at 37°C for 1 hour. Proteins were alkylated with 10 mM iodoacetamide and incubated at room temperature (RT) in the dark with shaking for 30 min. Samples were diluted into 6 ml 0.2 M Tris–HCl pH 8.0 to decrease the urea concentration to less than 2 M and to decrease the protein concentration. Trypsin (0.5 μg/μl, enzyme/protein ratio of 1/40 w/w) was added to digest the proteins at 30°C overnight. After digestion, 5 μg of sample was resolved on a tricine gel and silver staining was used to visualize the digestion products. The disappearance of high molecular bands and the appearance of a smear of bands with a molecular weight less than 10 kDa indicated the completion of digestion. The peptides were de-salted using a C18 cartridge according to the manufacturer’s instructions and then the peptides were oxidized by incubation with 10 mM sodium periodate in 80% ACN (v/v) 0.1% TFA (v/v) for 1 hour at RT in the dark. The oxidized peptides were purified by C18 cartridge, eluted in 80% ACN (v/v) 0.1% TFA (v/v) and mixed with 50 μl of (50% slurry) hydrazide support prewashed with 1 ml deionized water. The mixture was incubated with gentle shaking at RT overnight for the coupling reaction. The hydrazide support was washed three times with 800 μl of 1.5 M NaCl followed by three times with 800 μl of water to remove non-coupled peptides. The support was finally washed twice with 200 μl of 1× G7 buffer and incubated with 3 μl of PNGase F in 50 μl of 1× G7 buffer at 37°C to specifically release formerly N-glycosylated peptides which were purified by a C18 cartridge. Then, the peptides were dried in a speed-vac and re-suspended in 40 μl of 0.1% formic acid prior to MS analysis. For pooled glycopeptides, an equal volume (1 μl) of the formerly N-glycosylated peptides from each sample was pooled into either the HIV infected or uninfected pools.

### LC-MS/MS analysis

The formerly N-glycosylated peptides (1.5 μg) were separated using a Dionex Ultimate 3000 RELC nano system (Thermo Scientific) with a 75 μm × 15 cm Acclaim PepMap100 analytical column (Thermo Scientific) protected by a 2 cm guard column (Thermo Scientific). The mobile phase flow rate was 300 nl/min with 0.1% formic acid in water (A) and 0.1% formic acid/95% acetonitrile (B). The gradient profile was set as follows: 4-35% B for 70 min, 35-95% B for 5 min, 95% B for 10 min and equilibration at 4% B for 15 min. MS analysis was performed using an Orbitrap Velos Pro mass spectrometer (Thermo Scientific). The spray voltage was set at 2.2 kV. Orbitrap MS1 spectra (AGC 1×10^6^) were acquired from 400–1800 m/z at 60 K resolution followed by data-dependent HCD MS/MS (7,500 resolution, collision energy 45%, activation time 0.1 ms) of the ten most abundant ions using an isolation width of 2.0 Da. Charge state screening was enabled to reject unassigned and singly charged ions. A dynamic exclusion time of 35 sec was used to discriminate against previously selected ions.

### Protein identification and label-free quantitation

The quality of the Raw files from the LTQ-Orbitrap Velos Pro was evaluated by the Trans-Proteomic Pipeline (TPP) and searched against a human protein database (the International Protein Index human protein database, version 3.87). The precursor mass tolerance was 20 ppm and the MS/MS tolerance was 0.06 Da. The modification parameters of the database search were as follows: oxidized methionine (addition of 15.995 Da to Met), PNGase F-catalyzed conversion of Asn to Asp (addition of 0.984 Da to Asn) and Cys modification (addition of 57.021 to Cys). A maximum of two missed tryptic cleavage sites were used. The assigned peptides were evaluated by INTERACT and PeptideProphet with a minimum probability score of 0.7 [42-44]. Label-free quantitation was performed using MaxQuant ver. 1.3.0.5 and searched against the same human protein IPI database ver. 3.87 containing a total of 91,464 entries [38]. The default parameters with a 1% false discovery rate (FDR) were used except for the following: enzyme: trypsin; variable modifications: oxidation (M) and deamidation (NQ); and filter labeled amino acid unselected. The output peptide.txt file containing the identified peptide sequences and corresponding MS peak intensities were used for the quantitation.

### Data analysis

The output data from MaxQuant were converted to Microsoft Excel files. First, peptides that were considered contaminants, peptides that were identified in the reverse database search and peptides lacking N deamination were deleted. Second, peptides without an NXS/T (where X is any amino acid except proline) motif were deleted. The intensities of the remaining glycopeptides were normalized by dividing the total intensity of the identified glycopeptides in each LC-MS/MS run. The mean intensity of all the glycopeptides from a glycoprotein that had intensities greater than zero was used for inter-sample protein comparison. Using pooled samples, a cut-off ratio of 2-fold was used while a Student’s *t*-test with a *p-value* ≤ 0.05 was used to analyze the individual samples.

## Results

### Quantitative secretion of glycoproteins from T cells with HIV latent infection

**Figure 1:**
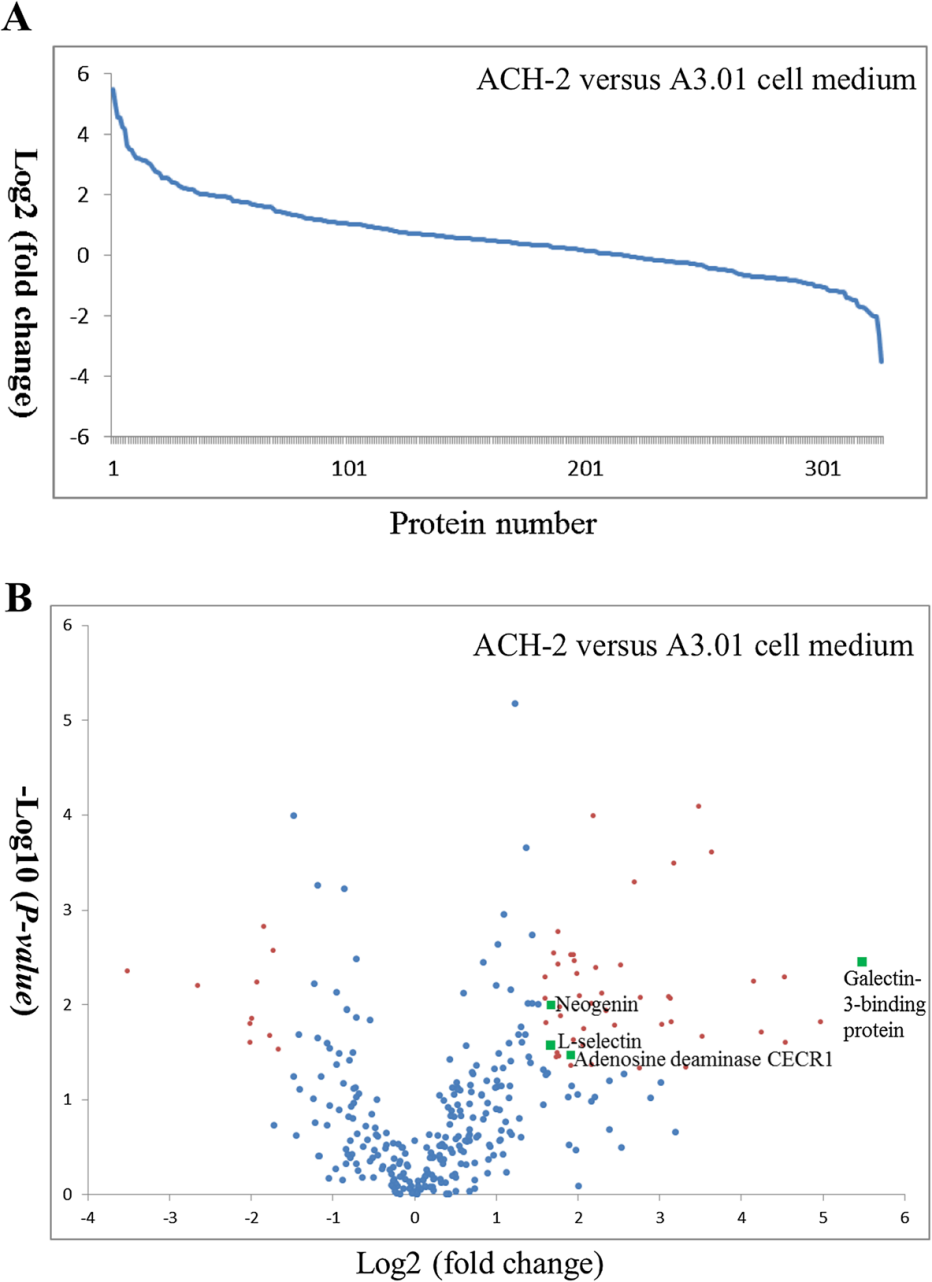
**Plots of the 326 proteins for label-free quantitative comparison between ACH-2 and A3.01 cell media. A)** Log_2_ fold-change plot of the proteins after normalization. **B)** Volcano plot of the proteins. Proteins with greater than a 3-fold change and *p-*value < 0.05 are colored in red and green. Green squares indicate proteins that were also increased in the HIV^+^ plasma sample.

To address whether HIV latent infection was associated with aberrant secretion of glycoproteins from T cells, serum-free culture media of HIV-1 latently infected T-lymphocyte ACH-2 cells and the parental cell line A3.01 cells were studied through the use of label free glycoproteomics. To evaluate the viral production of these two cultured cell lines, Western blotting-based immunoreactivity of gp120 and gp41 in ACH-2 cells stimulated by PMA was observed, but no immunoreactivity was detected in A3.01 cells was evident, demonstrating the status of latent infection in ACH-2 cells (Additional file 1: Figure S1). Serum-free medium of T cells was collected and processed using a SPEG-based method to isolate formerly N-glycosylated peptides as described in the Methods section. Subsequently, the reconstituted formerly glycosylated peptides from each sample were analyzed by three LC-MS/MS technical replicates. The quality of the reproducible LC-MS/MS data was inspected using Pep3D images generated from Trans Proteomic Pipeline (TPP) to evaluate retention time consistency and MS2 coverage (Additional file 2: Figure S2). TPP identified 829 unique glycopeptides from 411 unique glycoproteins with N deamidation within the consensus N-linked glycosylation motif (NXS/T, where X is any amino acid except proline). The quality of the data that were evaluated by TPP was adequate for downstream label-free comparison. Accordingly, MaxQuant was used to calculate the peak intensities, resulting in the identification of 671 glycopeptides from 375 glycoproteins. After normalization, 326 proteins could be quantitatively compared between the samples based on their fold-change and *p-*value (Figure 1). Using criteria of a fold-change of 3 and a *p-*value < 0.05, 59 glycoproteins were identified as being significantly differentially expressed (Figure 1B & Table 2).

**Table 2:** Differentially expressed proteins in the media of ACH-2 and A3.01 cells

**IPI ID**	**Protein name**	**Unique peptide #**	**Fold change**	***p-value***
IPI00023673	Galectin-3-binding proteins	6	44.6	4.E-03
IPI00437751	Angiotensin-converting enzyme	2	31.5	2.E-02
IPI00059476	Dipeptidase 1	3	23.3	3.E-02
IPI00010348	Deoxyribonuclease-2-alpha	1	23.1	5.E-03
IPI00171411	Golgi membrane protein 1	2	19.0	2.E-02
IPI00031802	Tumor necrosis factor ligand superfamily member 13B	1	17.8	6.E-03
IPI00472013	HLA class I histocompatibility antigen, A-33 alpha chain	1	12.4	2.E-04
IPI01013306	Highly similar to Homo sapiens L1 cell adhesion molecule	1	11.5	2.E-02
IPI00029419	Hereditary hemochromatosis protein	1	11.1	8.E-05
IPI00745313	Adipocyte enhancer-binding protein 1	2	10.0	5.E-02
IPI00784119	V-type proton ATPase subunit S1	1	9.1	3.E-04
IPI00797223	Serine protease 57	1	8.9	2.E-02
IPI00294834	Aspartyl/asparaginyl beta-hydroxylase	1	8.8	9.E-03
IPI00152540	CD109 antigen	7	8.7	8.E-03
IPI00299083	Junctional adhesion molecule B	1	8.2	2.E-02
IPI00298237	Tripeptidyl-peptidase 1	1	6.8	8.E-03
IPI00787853	Inositol monophosphatase 3	1	6.7	5.E-02
IPI00328113	Fibrillin-1	1	6.5	5.E-04
IPI00175654	Allergin-1	1	5.8	4.E-03
IPI00013744	Integrin alpha-2	2	5.5	2.E-02
IPI00217882	Sortilin	3	5.1	1.E-02
IPI00641181	MARCKS-related protein 1	1	4.9	8.E-03
IPI00176427	Cell adhesion molecule 4	1	4.7	4.E-03
IPI00789911	Lymphocyte antigen 75	9	4.5	1.E-04
IPI00151990	Thioredoxin domain-containing protein 15	1	4.5	1.E-02
IPI00553185	T-complex protein 1 subunit gamma	1	4.5	4.E-02
IPI00374563	Agrin	1	4.2	2.E-02
IPI00163187	Fascin	1	4.2	3.E-02
IPI00009923	Prolyl 4-hydroxylase subunit alpha-1	1	4.1	8.E-03
IPI00031801	DNA-binding protein A	1	4.0	5.E-03
IPI00290085	Cadherin-2	1	3.9	3.E-03
IPI00004084	Cyclic AMP-dependent transcription factor ATF-6 beta	1	3.9	2.E-02
IPI00022608	Sortilin-related receptor	7	3.9	3.E-03
IPI00001793	Beta-1,3-N-acetylglucosaminyltransferase radical fringe	1	3.8	4.E-02
IPI00301459	Group XV phospholipase A2	1	3.8	3.E-03
IPI00303071	Adenosine deaminase CECR1	2	3.8	3.E-02
IPI00983078	Transmembrane 9 superfamily member 1	1	3.5	1.E-02
IPI00980045	CD59 glycoprotein	1	3.4	1.E-02
IPI00020201	CMP-N-acetylneuraminate-poly-alpha-2,8-sialyltransferase	3	3.4	3.E-02
IPI00021794	Lysosomal protective protein	1	3.4	2.E-03
IPI00009629	CMP-N-acetylneuraminate-beta-galactosamide-alpha-2,3-sialyltransferase 1	3	3.4	4.E-03
IPI00797738	Cytochrome c oxidase subunit 6B1	1	3.4	3.E-02
IPI00000070	Low-density lipoprotein receptor	1	3.3	4.E-02
IPI00556494	Mediator of RNA polymerase II transcription subunit 4	1	3.3	3.E-03
IPI00023814	Neogenin	2	3.2	1.E-02
IPI00218795	L-selectin	2	3.2	3.E-02
IPI00893133	MHC class I antigen	1	3.1	2.E-02
IPI00031821	Integral membrane protein 2B	1	3.0	5.E-03
IPI00465261	Endoplasmic reticulum aminopeptidase 2	1	3.0	9.E-03
IPI00168728	FLJ00385 protein	1	-3.2	3.E-02
IPI00299594	Neuropilin-1	1	-3.3	3.E-03
IPI00015476	Neutral amino acid transporter A	1	-3.4	2.E-02
IPI00029739	Complement factor H	2	-3.6	1.E-03
IPI00433138	Discoidin, CUB and LCCL domain-containing protein 2	1	-3.8	6.E-03
IPI00000736	Tetraspanin-15	1	-4.0	1.E-02
IPI00183782	T-lymphocyte surface antigen Ly-9	2	-4.0	2.E-02
IPI00022558	Myelin protein zero-like protein 1	1	-4.0	2.E-02
IPI00216569	Cystatin-F	2	-6.3	6.E-03
IPI00328431	Netrin receptor UNC5B	1	-11.5	4.E-03

### Elevated plasma glycoprotein might be influenced by HIV latently infected T cells

**Figure 2:**
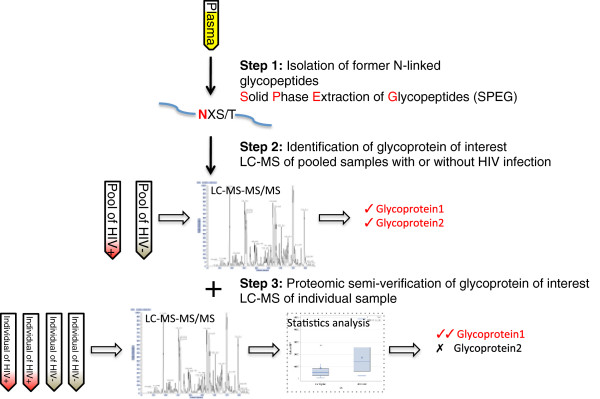
**Strategy used for the identification of differentially expressed glycoproteins in HIV**^**+ **^**human plasma.**

To determine whether proteins secreted by HIV latently infected T cells could be detected in HIV^+^ plasma, 20 plasma samples from 10 HIV^+^ and 10 HIV^-^ patients were evaluated. Although the time since HIV infection was not known, it was possible to assume that all the HIV^+^ samples were not recently infected according to the clinical observation in the HPTN lab. In theory, these plasma samples could harbor proteins secreted from T cells with HIV latent infection. A schematic flow chart of the approach used to study the changes in glycoprotein relative abundance is shown in Figure 2. The 20 plasma samples were processed individually using SPEG-glycoproteomics and pooled as described in the Methods section. The pooled samples were used to screen the glycoproteins between these two groups. Label-free quantification was used to analyze data from 4 consecutive LC-MS/MS runs for the HIV^+^ pool and 3 LC-MS/MS runs for the HIV^-^ pool. The quality of the LC-MS/MS data was again evaluated using Pep3D images (Additional file 3: Figure S3). Eight hundred fifty unique glycopeptides from 221 unique glycoproteins were identified by TPP (data not shown). MaxQuant analysis identified 399 unique glycopeptides from 183 glycoproteins (data not shown). Of these proteins, 103 glycoproteins were quantitatively compared between the HIV^+^ and HIV^-^ groups using a cut-off ratio of 1.3-fold (data not shown). To determine whether these proteins were quantitatively different in individuals from each group, an equal volume (1 μl per LC-MS/MS injection) of the 20 individual plasma samples was analyzed by LC-MS/MS with 3 technical replicates for each plasma sample followed again by TPP quality evaluation and MaxQuant analysis (588 unique glycopeptides from 253 glycoproteins were identified). This step semi-verified and reduced the total number of differentially expressed proteins from 103 to 38 using a *p-*value < 0.05 and a cut-off ratio of 1.5-fold (Table 3). A comparison between the differential 38 proteins in the plasma and the 59 proteins detected in the T cell study revealed that four proteins (galecin-3-binding protein, L-selectin, neogenin and adenosine deaminase CECR1) were identified in both analyses as having a greater than 3-fold increased relative abundance in the medium of ACH-2 cells and a 2-fold increased relative abundance in HIV^+^ plasma (Table 3). Noticeably, compared to A3.01 cells, galectin-3-binding protein demonstrated the most significant upregulation which was more than 45-fold increased compared to the level that was detected in the medium of ACH-2 cells (Table 3). In addition, when the 38 proteins in plasma were searched against the 326 proteins observed in the T cell medium study, two proteins, phospholipid transfer protein and ICOS ligand, were found to be increased by 5.1- and 2.6-fold, respectively, although the former had a *p-*value slightly greater than 0.05 in the T cell study (Table 3). The elevated levels of these six proteins in the medium of ACH-2 cells and HIV^+^ plasma suggested that their up-regulation in the HIV^+^ plasma might be partially due to HIV latently infected T cells.

**Table 3:** **N-linked glycoprotein changes in the plasma of HIV**^**+ **^**and HIV**^**- **^**patients and their changed levels in T cells with HIV latent infection**

**IPI ID**	**Protein name**	**Individual analysis (HIV+/HIV-)**			**Medium (ACH-2/A3.01)**		
		**Unique peptide #**	**Fold change**	***P-value***	**Unique peptide #**	**Fold change**	***P-value***
IPI00759642	Scavenger receptor cysteine-rich type 1 protein M130	3	4.7	2.E-03	-	-	-
IPI00029756	Tyrosine-protein kinase Mer	2	3.8	2.E-02	-	-	-
IPI00008494	Intercellular adhesion molecule 1	3	3.0	1.E-02	2	NS	NS
IPI00023814	Neogenin	4	2.9	3.E-03	2	3.2	1.E-02
IPI00009477	Intercellular adhesion molecule 2	5	2.9	2.E-06	5	NS	NS
IPI00242956	IgGFc-binding protein	12	2.9	1.E-07	-	-	-
IPI00017841	Noelin	4	2.9	4.E-02	-	-	-
IPI00004573	Polymeric immunoglobulin receptor	6	2.8	2.E-03	-	-	-
IPI00011229	Cathepsin D	1	2.8	6.E-03	2	NS	NS
IPI00290328	Receptor-type tyrosine-protein phosphatase eta	3	2.7	5.E-03	1	NS	NS
IPI00290856	Lymphatic vessel endothelial hyaluronic acid receptor 1	2	2.7	1.E-02	-	-	-
IPI00437186	Probable G-protein coupled receptor 116	4	2.6	1.E-02	-	-	-
IPI00166392	Cell adhesion molecule 1	3	2.5	2.E-02	-	-	-
IPI00015102	CD166 antigen	1	2.5	3.E-03	4	NS	NS
IPI00297124	Interleukin-6 receptor subunit beta	1	2.4	3.E-02	2	NS	NS
IPI00303071	Adenosine deaminase CECR1	2	2.4	1.E-02	2	3.8	3.E-02
IPI00023673	Galectin-3-binding protein	7	2.3	3.E-07	6	44.5	4.E-03
IPI00218795	L-selectin	3	2.2	1.E-02	2	3.2	3.E-02
IPI00643034	Phospholipid transfer protein	4	2.1	2.E-03	3	5.9	5.E-02
IPI00018136	Vascular cell adhesion protein 1	4	2.0	2.E-02	-	-	-
IPI00478003	Alpha-2-macroglobulin	6	2.0	3.E-03	-	-	-
IPI00297263	Protein HEG homolog 1	2	1.9	2.E-02	-	-	-
IPI00889740	Fibulin 1	2	1.9	4.E-02	-	-	-
IPI00023014	Von Willebrand factor	9	1.8	3.E-02	-	-	-
IPI00896380	Ig mu chain C region	5	1.7	5.E-02	-	-	-
IPI00020986	Lumican	5	1.7	2.E-02	-	-	-
IPI00414888	ICOS ligand	3	1.7	5.E-02	2	2.6	1.E-02
IPI00022462	Transferrin receptor protein 1	3	1.6	3.E-04	4	NS	NS
IPI00973474	Ig gamma-3 chain C region	2	1.6	2.E-02	-	-	-
IPI00009521	Macrophage receptor MARCO	1	1.5	4.E-02	-	-	-
IPI00011264	Complement factor H-related protein 1	1	-1.5	4.E-02	-	-	-
IPI00011252	Complement component C8 alpha chain	1	-1.5	4.E-02	-	-	-
IPI00879573	Heparin cofactor 2	5	-1.6	2.E-03	-	-	-
IPI00641737	Haptoglobin	6	-1.6	3.E-02	-	-	-
IPI00299778	Serum paraoxonase/lactonase 3	1	-1.6	2.E-03	-	-	-
IPI00028413	Inter-alpha-trypsin inhibitor heavy chain H3	1	-1.7	3.E-02	-	-	-
IPI00299435	Apolipoprotein F	1	-2.0	2.E-04	-	-	-
IPI00027507	Complement factor H-related protein 3	3	-2.7	2.E-03	-	-	-

Plasma (n=20) was analyzed individually by glycoproteomics. The protein fold changes and p-values were calculated as described in the Methods. After 48 hours of culture, the serum-free media of the HIV latently infected T cell line ACH-2 and its parental cell line A3.01 were compared by glycoproteomics as described in the Methods. NS - Not Significant.

## Discussion

The identification of protein changes in the plasma of patients infected with HIV could lead to the identification of biomarkers that have roles in pathogenesis and that have clinical applications. However, as an important area of the proteomic study of post translational modifications (PTM), glycoproteomics has not been applied in HIV studies. One of the main concerns is whether the identified change of protein abundance in the patients is specific to HIV infection. In an attempt to address this question, we studied *in vitro* T-cells with latent HIV (i.e. T cells with HIV DNA integrated into the genome of the cell but not producing virions) to identify the glycoproteins that are preferentially secreted from this type of T cells. The stage of HIV DNA integration in the viral life cycle is particularly important because it occurs prior to latent and productive infection. Presumably, the productive infection of HIV to a large extent would trigger more intensive host responses than latent infection. However, the intracellular material from dying cells and the intense host response due to productive infection might result in the detection of an abundance of dysregulated proteins. In contrast, fewer dysregulated proteins would, presumably, be secreted silently during the latent stage of T cells. These secreted proteins could constantly modulate the host immune system due to the longer survival time of the cells. These cells also remain HIV replication-competent for potential invasion of the immune system.

One drawback of using a transformed cell line is that it does not always function similarly to native infected CD4+ primary cells that would commonly be used for future data validation. The identified glycoproteins from T cells media provide a list of protein candidates that may have a regulatory role in host-HIV interaction. The association between the T cell-secreted glycoproteins and HIV infection *in vivo* was then analyzed using HIV^+^ plasma. Based on the viral load data (Table 1), the patient population likely represented latent and active viral replication. It is worth noting that HIV latent infection in T cells persisted even when viral replication was active. The purpose of using patient plasma was to determine whether the glycoproteins identified from the T cell medium could be detected and whether they were differentially expressed in HIV infection in general. This study of HIV infected patient plasma resulted in the identification of six proteins that were also present in the T cell media. Although the specificity of the molecules to HIV infection is not conclusive and requires further determination, this first application of glycoproteomics to HIV latently infected T cells and HIV^+^ plasma elucidated significantly higher levels of glycoproteins that could aid future studies that are designed to understand host-HIV interaction *in vivo*.

HIV latent infection could affect the protein T cell protein secretion, and the secreted proteins could be potent immuno-modulators. We hypothesized that the secretion or leaking of protein from these HIV infected T cells might contribute to the dysregulation of glycoproteins in the plasma of HIV-infected patients. To evaluate this possibility, ACH-2 and its parental A3.01 cell line were studied because of their ability to mimic the latent infection of HIV, although these cell lines behave differently from canonical peripheral blood mononuclear cell (PBMC) in many aspects. Nevertheless, the T cell lines were homogeneous and they provided direct evidence supporting the proposed hypothesis. Our glycoproteomic study of the media from these T cells identified 59 glycoproteins with at least a 3-fold change and *p-*value < 0.05. These glycoproteins could be of interest because their increased levels might have a regulatory effect in the host response. Next, to determine whether any of the changes in protein levels in the T cell media were also detected in the plasma from HIV^+^ patients, we employed a strategy that used pooled samples for the initial screen and individual samples for the downstream high throughput verification. The use of this strategy is thought to solve some of the issues associated with ELISA testing (i.e. relatively poor sensitivity of detection and lack of cost effectiveness in evaluating a large number of proteins). Although the strategy limited the sample size here, it is noteworthy that a strategy could be developed that begins with the analysis of pooled samples and is then followed by verification using a predetermined number of randomly selected samples from the pools.

The analysis of pooled samples identified 103 glycoproteins between the HIV^+^ and HIV^-^group with a cut-off ratio of 1.3-fold. The low cut-off ratio retained as many proteins as possible in the list for subsequent MS verification. The 20 samples were then analyzed individually by three technical replicates and the results indicated that 38 proteins were differentially expressed between groups. The analysis of individual samples permitted the statistical evaluation (*p-*value ≤ 0.05) of each protein in the list. A fold change in the same trend observed in the analysis of the pooled and individual samples provided a means to verify these proteins. These 38 proteins accounting for 36.9% of the total 103 proteins suggested that HIV infection has a moderate effect on glycoproteins, although it was not clear whether the effect was on protein expression or the glycosylation level of the changed glycoproteins.

Among these 38 proteins, four proteins – galectin-3-binding protein, L-selectin, neogenin, and phospholipid transfer protein – were determined to have an increase of at least 3-fold in the medium of virus latently infected T cells and a 2-fold increase in HIV^+^ plasma. Galectin-3-binding protein was the most significantly up-regulated protein in the medium of ACH-2 cells. Although galectin-3-binding protein has been reported to be elevated in HIV infection and cancer [45], the cause of its elevation in either disease is unclear *in vivo*. The up-regulation of galectin-3-binding protein in the medium of ACH-2 cells suggested that T cells latently infected with HIV could contribute to the elevation of galectin-3-binding protein that was observed in the plasma of HIV^+^ patients. Interestingly, an increase of L-selectin in HIV infection has been reported to function in the homing of T lymphocytes to the peripheral lymph nodes for apoptosis [46-48]. This mechanism possibly explains the massive loss of these cells without obvious intracellular HIV replication [46-48]. Shedding of L-selectin is reported to be induced through the engagement of HIV to both CD4 and CXCR4 [48]. Our results indicate that HIV latency could result in the secretion of L-selectin as well. It is more likely that the dysregulation of proteins in the plasma is a consequence of an orchestrated series of host responses.

Four proteins – neogenin, adenosine deaminase CECR1, ICOS ligand and phospholipid transfer protein – may also participate in host-HIV interaction. **Neogenin**, which belongs to the immunoglobulin superfamily, is a multifunctional transmembrane receptor. In neogenin^-/-^ animals, significant attenuation of induced inflammation has been observed, and, interestingly, direct injection of anti-neogenin antibody in animals also significantly reduces inflammation [49,50]. These studies suggest that neogenin may play an important role in chronic inflammation induced by HIV infection. Elevated levels of neogenin were observed by MS analysis in our study. Hence, functional regulation of its level may have a therapeutic value in HIV^+^ patients. **Adenosine deaminase CECR1** functions to degrade extracellular adenosine, and it has been reported to increase the proliferation rate of CD4 T cells and macrophages independent of its enzyme activity [51]. The increased proliferation of these cell populations with a portion of them harboring HIV latency may result in the expansion of the HIV reservoir. **ICOS ligand** and the ICOS pathway function to trigger effector T cell responses and generate follicular helper T cells [52]. HIV infection severely affects the effector T cell population [53]. Prolonged activation of effector T cells by ICOS ligand could lead to progressive exhaustion. In addition, a recent study reported that the main CD4 T cell compartment for HIV is follicular helper T cells [54]. Thus, excessive ICOS ligand may stimulate the generation of such cells whose increase in number supports future HIV infection and replication. **Phospholipid transfer protein** functions in lipoprotein metabolism and lipid transport in the vascular compartment. Its levels could be changed in different human diseases and conditions [55]. Our finding of elevated phospholipid transfer protein levels could be a result of overall immune activation or inflammatory reaction triggered by HIV chronic infection. Aside from the four proteins mentioned above, other proteins among those listed in Tables 2 &3 may be of interest in regard to their potentially important roles in HIV infection. A careful selection of the proteins merits future investigation.

## Conclusion

To our knowledge, this study is the first application of glycoproteomics in HIV latently infected T cell medium and HIV^+^ plasma. We identified several glycoproteins with differential expression in the medium of an HIV infected T cell model and HIV^+^ plasma. The correlation of the proteins observed in the medium of HIV^+^ T cells and plasma was not clear in the study. To specifically associate the changed glycoproteins with HIV infection, the analysis of clearly defined patient cohorts merits future glycoproteomic investigation.

## Competing interests

The authors declare that they have no competing interests.

## Authors’ contributions

WY and SS carried out the cell culture and sample preparation. JZ carried out the mass spectrometry. WY, LC, MA and PA analyzed the MS data. AS prepared the HIV clinical sample and information. WY, HZ and JBJ designed the experiment and drafted the manuscript. All authors read and approved the final manuscript.

## Supplementary Material

Additional file 1: Figure S1 Confirmation of HIV latent infection in ACH-2 cells stimulated by PMA for 48 hours. Click here for file

Additional file 2: Figure S2 Pep3D image of LC-MS replicate analyses of ACH-2 and A3.01 media. Click here for file

Additional file 3: Figure S3 Pep3D image of LC-MS replicate analyses of HIV+ and HIV- pooled samples. Click here for file
