# Supplementary figures and images for: Glycoproteomic analysis identifies human glycoproteins secreted from HIV latently infected T cells and reveals their presence in HIV+ plasma
Source: Clin Proteomics. 2014 Mar 6;11(1):9. doi: 10.1186/1559-0275-11-9 (PMC4015807; doi:10.1186/1559-0275-11-9)

Figure S1: Confirmation of HIV latent infection in ACH-2 cells stimulated by PMA for 48 hours.


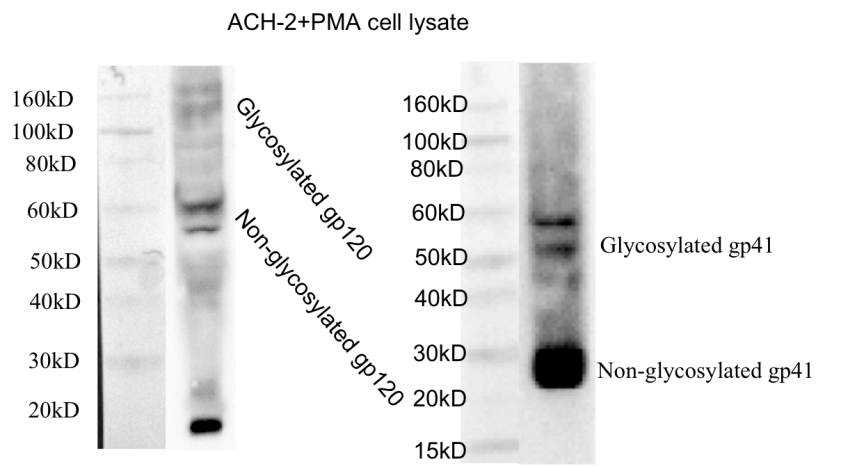

Supplement: Additional file 1: Figure S1 — Confirmation of HIV latent infection in ACH-2 cells stimulated by PMA for 48 hours. [file 1559-0275-11-9-S1.doc]

Figure S2: Pep3D image of LC-MS replicate analyses of ACH-2 and A3.01 media.


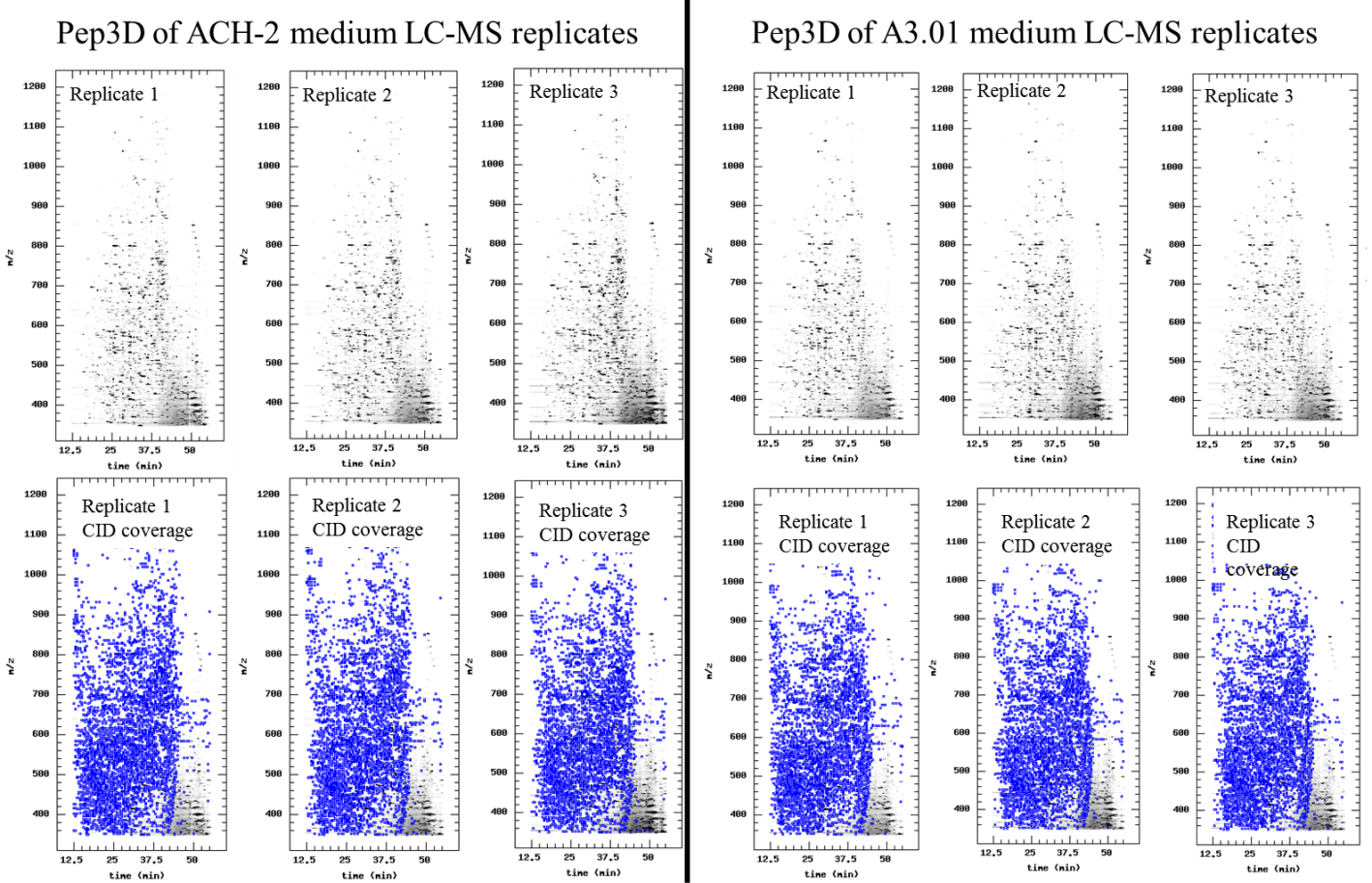

Supplement: Additional file 2: Figure S2 — Pep3D image of LC-MS replicate analyses of ACH-2 and A3.01 media. [file 1559-0275-11-9-S2.doc]

Figure S3: Pep3D image of LC-MS replicate analyses of HIV+ and HIV- pooled samples.


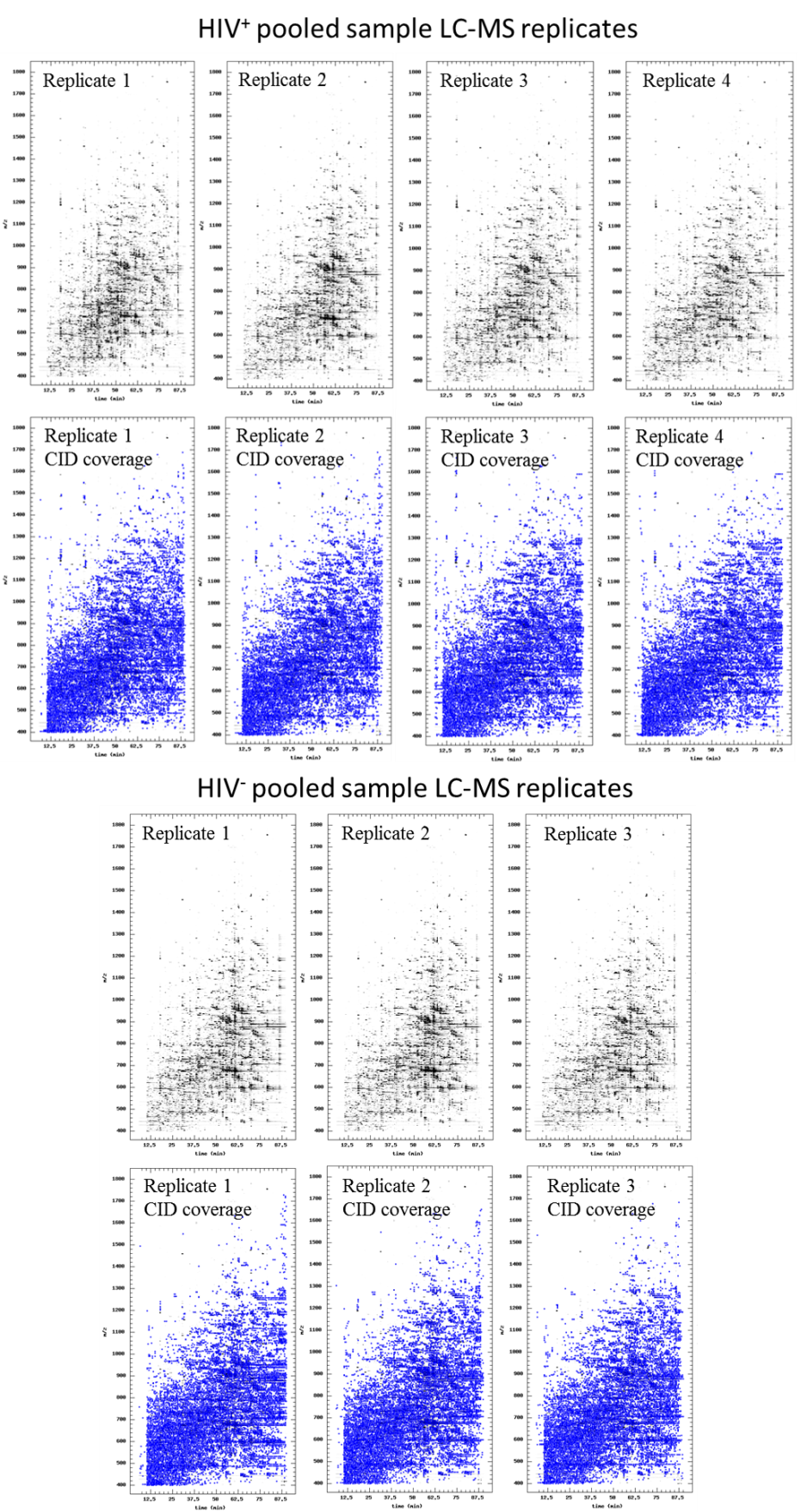

Supplement: Additional file 3: Figure S3 — Pep3D image of LC-MS replicate analyses of HIV+ and HIV- pooled samples. [file 1559-0275-11-9-S3.doc]
